# Identification and Validation of an Explainable Predictive Model For Heart Failure in Patients With Hypertension

**DOI:** 10.31083/RCM46901

**Published:** 2026-05-21

**Authors:** Jiayi Han, Tengxiao Zhao, Yuncong Shi, Zehao Zhao, Wenjie Wang, Yi Ye, Yingxuan Bai, Zhihan Lin, Xiangfei Meng, Liwei Guo, Ruixiang Feng, Yaodong Ding, Yong Zeng

**Affiliations:** ^1^Department of Cardiology, Center for Coronary Artery Disease, Beijing Anzhen Hospital, Capital Medical University, 100029 Beijing, China

**Keywords:** heart failure, hypertension, machine learning, risk factors, cohort studies, predictive value of tests

## Abstract

**Background:**

Heart failure (HF) is a heterogeneous syndrome affecting over 60 million individuals globally. Patients with hypertension are particularly susceptible to developing HF. Therefore, timely identification and predictive assessment of HF risk have significant clinical implications in this population. Thus, this study aimed to develop a new interpretable machine learning (ML) model for HF prediction.

**Methods:**

Using data from the Systolic Blood Pressure Intervention Trial (SPRINT), a random under-sampling technique was applied to address class imbalance in the target variable, achieving a 1:1 ratio between positive and negative samples. By randomly matching 162 individuals without HF events to those with events, a balanced dataset comprising 324 participants was constructed. The test set comprised 40% of the total dataset to ensure a robust evaluation of model performance. Seven ML algorithms, including support vector machine (SVM), adaptive boosting (Adaboost), naïve Bayes (NB), logistic regression (LR), gradient boosting machine (GBM), random forest (RF), and multilayer perceptron (MLP), were employed to construct the predictive models. Model performance was evaluated using the area under the curve (AUC), decision curve analysis (DCA), calibration curves, and other metrics. The SHapley Additive exPlanations (SHAP) approach was employed to rank feature significance and provide interpretability for the final model.

**Results:**

Over a median follow-up of 3.88years, 162 patients (1.8%) developed incident HF. Among the seven ML models, GBM demonstrated the best performance. A total of 14 features were retained after the least absolute shrinkage and selection operator (LASSO) selection. The final model exhibited robust predictive capability for identifying HF risk, with an overall accuracy of 0.731, a precision of 0.770, and an AUC (95% confidence interval (CI)) of 0.763 (0.676–0.840).

**Conclusion:**

The GBM-based explainable prediction model demonstrated robust performance in predicting HF risk among patients with hypertension.

## 1. Introduction

Hypertension is a high risk factor for cardiovascular disease [1]. Elevated diastolic blood pressure (DBP), and especially systolic blood pressure (SBP), are strongly implicated in the development of heart failure (HF) [2]. One of the most significant observations from the Framingham cohort study was that the cumulative incidence of HF was markedly higher in patients with hypertension [3, 4, 5]. Studies have demonstrated that the prevention and treatment of hypertension significantly reduce the incidence of heart failure [5]. Hence, early and accurate identification of patients with hypertension at high risk for HF is critical for timely intervention and improved clinical outcomes.

Currently, a gap remains in the prediction of HF risk among individuals with hypertension. The majority of studies focused on constructing heart failure prediction models based on normal populations [6, 7, 8, 9]. Segar *et al*. [10] applied machine learning models to diabetic cohorts, and Yang *et al*. [11] focused on cancer patients. Katz *et al*. [12] pioneered the application of an unsupervised machine learning method, unbiased machine learning of dense phenotypic data (“phenomapping”), to hypertensive populations, demonstrating its predictive value for heart failure. However, this approach exhibits limited predictive utility for heart failure compared to supervised learning methods and demonstrates poor performance in reflecting relationships between clinical characteristics and outcomes.

Machine learning (ML), a critical component of artificial intelligence, has demonstrated remarkable capability in processing extensive datasets and accurately identifying complex patterns [13]. By emulating the data processing capabilities of the human brain, ML attains significantly higher accuracy and efficiency compared with traditional methods [14]. Currently, machine learning models used to predict heart failure in individuals with diabetes and cancer have demonstrated relatively high accuracy [10, 11, 15]. Nevertheless, despite their complexity, ML methods remain constrained by their lack of interpretability, often referred to as the “black-box” problem [16]. The SHapley Additive exPlanation (SHAP) method is a commonly used approach to overcome the “black-box” problem [16]. To date, no studies have investigated the use of supervised machine learning or other superior methods to predict heart failure risk in hypertensive populations. Furthermore, no existing studies have integrated intensified blood pressure control measures, hypertensive cohort data, and SHAP-based interpretability into models for predicting HF.

In this context, the present study developed and validated explainable ML models for early and accurate prediction of HF in patients with hypertension from the Systolic Blood Pressure Intervention Trial (SPRINT).

## 2. Methods

**Fig. 1.**
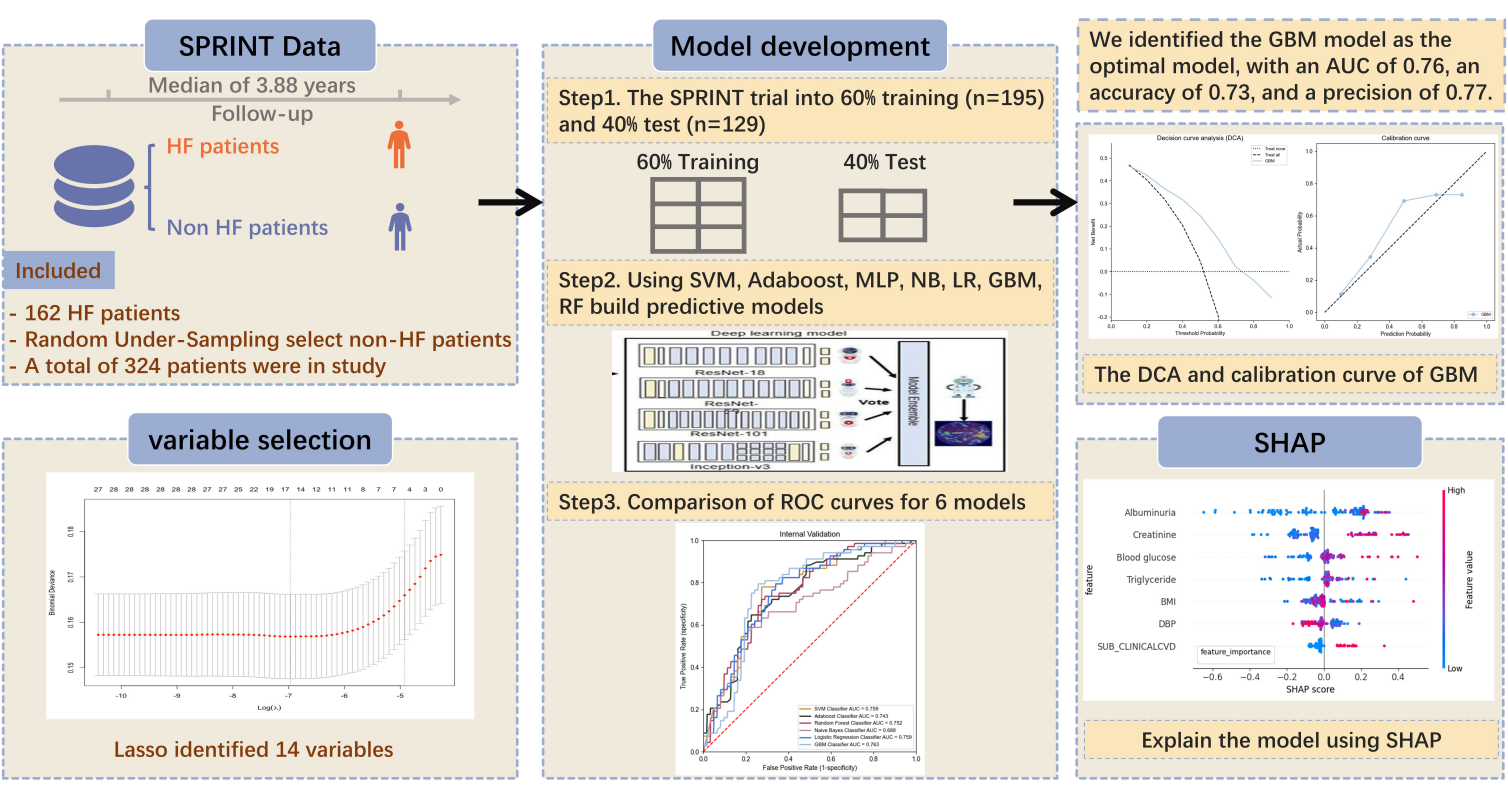
**Construction of machine learning models for predicting heart failure in hypertensive patients**. HF, heart failure; SPRINT, Systolic Blood Pressure Intervention Trial; SVM, support vector machine; Adaboost, adaptive boosting; MLP, multilayer perceptron; NB, naive Bayes; LR, logistic regression; GBM, gradient boosting machine; RF, random forest; AUC, area under curve; DCA, decision curve analysis; SHAP, SHapley Additive exPlanations; ROC, receiver operating characteristic.

The study design is shown in Fig. 1.

### 2.1 Systolic Blood Pressure Intervention Trial (SPRINT)

The relevant details of the SPRINT trial have been reported in previous studies [17, 18]. Briefly, the SPRINT study included a total of 9361 patients without diabetes, aged ≥50 years, and with a SBP of 130–180 mmHg. Patients were randomly assigned to the intensive treatment (SBP <120 mmHg) or standard treatment (SBP <140 mmHg) group. All patients had elevated risk of cardiovascular disease owing to advanced age (>75 years), presence of chronic kidney disease (CKD), established cardiovascular disease (excluding stroke), or a Framingham risk score >15% over 10 years. The intensive treatment group attained a mean SBP of 121.4 mmHg, compared to 136.2 mmHg in the standard treatment group [17, 19]. All participants provided informed consent to participate in this study.

### 2.2 Definition of HF

The HF outcome was defined as an adjudicated event of hospitalization or an emergency department visit for acute decompensated HF requiring intravenous therapy. Case adjudication was performed according to the Atherosclerosis Risk in Communities (ARIC) study protocol [20]. This outcome included both “definite” and “possible” cases of acute decompensated HF, with either preserved or reduced ejection fraction. Classification was based on a holistic review of multiple clinical sources, guided by pre-specified criteria and clinical judgment, rather than any single data point. “Possible” HF was assigned when decompensation could not be definitively distinguished from a concurrent comorbidity [21].

### 2.3 Data Processing

Based on relevant clinical experience, we extracted 28 clinical variables from the SPRINT database, specifically: Intensive blood-pressure treatment, Framingham estimation of 10-year cardiovascular disease‌ (CVD) risk, Framingham 10-year CVD risk >15% (INCLUSIONFRS), SBP, seated DBP, number of anti-hypertensive medications prescribed (N_AGENTS), participants on no anti-hypertensive agents, smoking (SMOKE_3CAT), aspirin use, epidermal growth factor receptor (eGFR), serum creatinine, subgroup with CKD (eGFR <60 mL/min/1.73 m^2^) (SUB_CKD), race (Black, African-American) (RACE_BLACK), age, gender, subgroup with history of clinical/subclinical CVD (SUB_CVD), subgroup with history of clinical CVD (SUB_CLINICALCVD), subgroup with history of subclinical CVD, subgroup ≥75 years old at randomization, race (White, Hispanic, Black, other) (RACE4), cholesterol, glucose, high-density lipoprotein (HDL), triglycerides, UMALCR [Urine Albumin/Creatinine ratio - mg Urine Alb / (g Creat * 0.01)], body mass index (BMI), statin use, systolic blood pressure tertile.

**Fig. 2.**
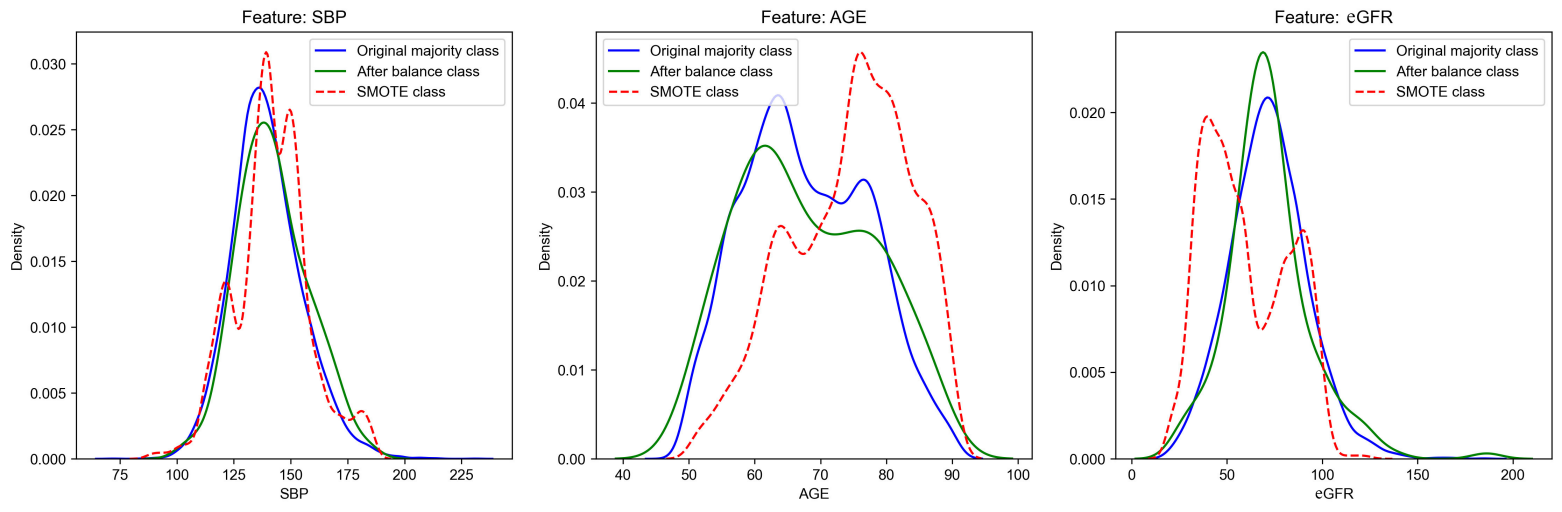
**The distribution of some core features after balancing**. SMOTE: Synthetic Minority Oversampling Technique, a composite sampling algorithm for synthesizing artificial data. SBP, systolic blood pressure; eGFR, epidermal growth factor receptor.

All variables with a missing rate exceeding 20% have been excluded. The remaining numerical variables were imputed using median imputation. Categorical variables contained no missing values. The substantial imbalance between positive and negative samples in the original dataset may bias the model toward the majority class, thereby diminishing its capacity to correctly identify positive samples. To mitigate this class imbalance in the target variable, a random under-sampling approach was applied to balance the positive and negative samples at a 1:1 ratio. The distribution and mean of core features before and after undersampling were verified to be largely consistent with the feature distribution of the original data, whereas certain weighted sampling methods may cause alterations in the data characteristics (Fig. 2). Therefore, by randomly matching 162 individuals without HF endpoint events to those with events, a balanced dataset comprising 324 participants was constructed. This approach is intended to direct the model’s focus toward minority class characteristics during training, thereby enhancing its predictive performance.

The balanced data was divided into a training set and a validation set in a 6:4 ratio. We trained the model by using the training set and used the validation set to simulate its generalization ability on unseen data. All continuous variables were standardized using the StandardScaler to obtain Z-scores (mean = 0, variance = 1), thereby adapting to the distribution characteristics of clinical data.

### 2.4 Model Development and Comparison

**Fig. 3.**
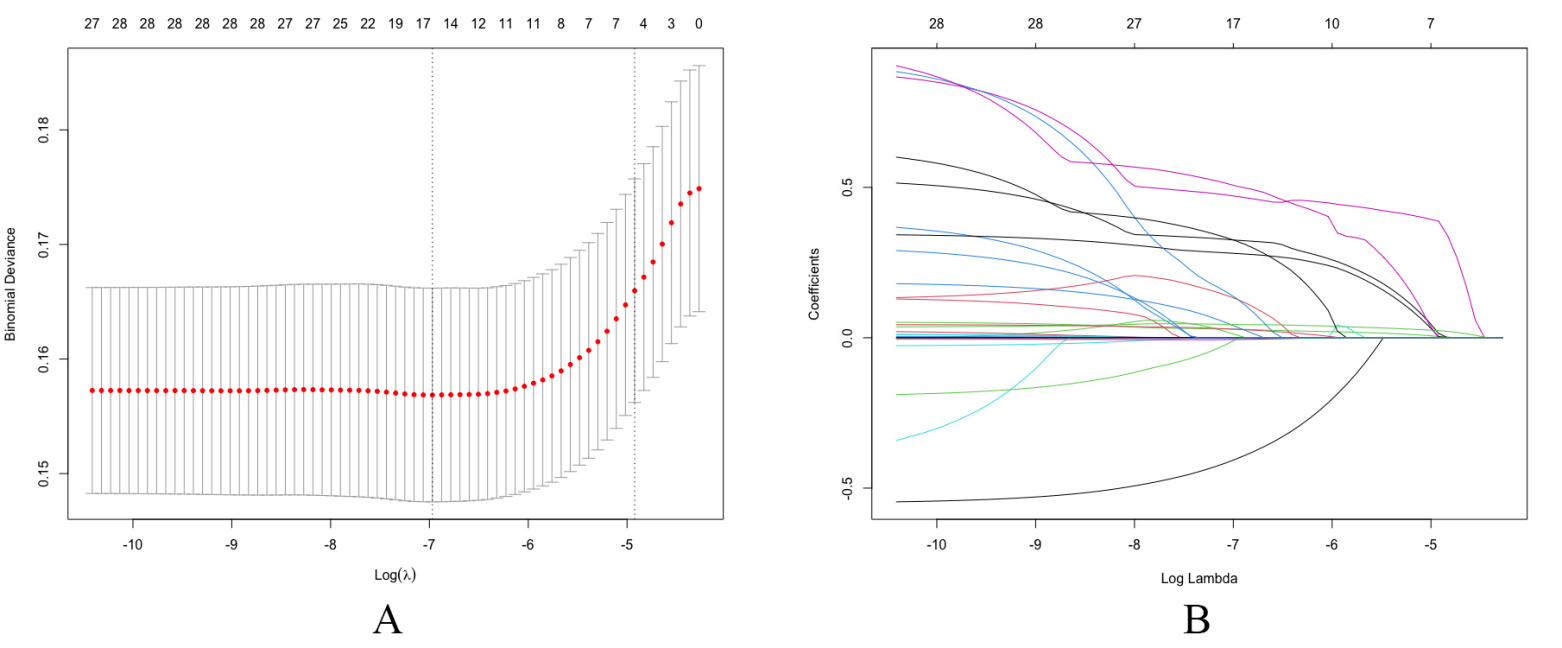
**Feature selection using the LASSO regression model**. (A) LASSO cross-validation. Key variables were identified using the LASSO regression algorithm and subsequently incorporated into the final model, followed by ten-fold cross-validation. According to the turning points, 14 variables were included in the study. (B) LASSO coefficient profiles of variables. LASSO, least absolute shrinkage and selection operator.

The least absolute shrinkage and selection operator (LASSO) is a regularization method that reduces feature coefficients, thereby enhancing the model’s generalization. The principal features were selected using LASSO regression with an optimal lambda value of 0.007354, determined solely from the training set. Ten-fold cross-validation was adopted to ensure model stability. The LASSO cross-validation and coefficient are shown in Fig. 3.

Based on the selected variables, prediction models were constructed using support vector machine (SVM), adaptive boosting (Adaboost), multilayer perceptron (MLP), naive Bayes (NB), logistic regression (LR), gradient boosting machine (GBM), and random forest (RF) algorithms. Grid search was used to optimize parameters for each algorithm. An LR model was constructed using the selected variables and further refined through 10-fold cross-validation. Model performance and clinical utility of each model were evaluated using decision curve analysis (DCA) and calibration curves, and the optimal model was subsequently selected. Repeated 10-fold cross-validation was employed to demonstrate that the model’s performance is both reliable and stable. Within the training set, 10-fold cross-validation was employed to verify the reliability and stability of all the models.

The area under the curve (AUC), sensitivity, specificity, accuracy, precision and F1 score were used to evaluate the reliability of these models. In addition, a calibration curve was utilized to assess the agreement between the predicted probabilities and the observed event rates.

### 2.5 Feature & Model Explanation

Interpreting ML models remains challenging due to their inherent complexity. To explain the machine learning “black box” nature, the SHAP method was used to rank feature importance and interpret model predictions [22].

SHAP values quantify the contribution of each feature to a specific prediction: positive values indicate an increased predicted risk, while negative values suggest decreased risk. The greater the absolute value, the stronger the feature’s influence.

SHAP supported both global and local interpretability. At the global level, it generated consistent and accurate attribution scores for each feature, revealing relationships between input variables and HF. At the local level, it enabled individualized explanation of predictions based on specific input data.

### 2.6 Statistical Analysis

Data analysis was performed by using Python (version 2.7.17; Python Software Foundation, Wilmington, DE, USA), with the pandas library for data manipulation, SHAP for model interpretability, and scikit-learn for ML tasks. The Mann-Whitney U test was used to compare continuous variables, while Fisher’s exact test or the χ^2^ test for categorical variables, depending on data distribution and sample size. The DeLong test was used to compare the AUC of the models.

## 3. Results

### 3.1 Patient Characteristics

A post hoc analysis of the SPRINT study, including 9361 patients with hypertension, was performed to develop a predictive model for HF, with a median follow-up of 3.88 years. Overall, 162 participants (cumulative incidence: 1.8%) developed HF. Compared to those without HF, participants who experienced incident HF events are older, have lower DBP and eGFR, higher albuminuria and a higher prevalence of CVD and CKD (all *p* < 0.05) (Table 1).

**Table 1.** **No HF event vs. HF event participants’ baseline characteristics**.

		No HF event	HF event	*p*
Age (mean (SD))		67.9 (10.2)	74.4 (9.9)	<0.001
DBP (mean (SD))		79.5 (11.8)	73.4 (13.2)	<0.001
SBP (mean (SD))		142.2 (15.5)	141.4 (17.7)	0.640
eGFR (mean (SD))		72.1 (21.9)	60.0 (22.7)	<0.001
Cholesterol (mean (SD))		189.2 (37.1)	183.4 (40.9)	0.185
HDL (mean (SD))		51.6 (14.4)	51.0 (14.0)	0.687
Albuminuria (mean (SD))		27.87 (57.24)	171.97 (521.56)	0.001
BMI (mean (SD))		29.4 (5.0)	30.1 (6.3)	0.268
FEMALES, n (%)				0.480
	0	105 (64.8)	111 (68.5)	
	1	57 (35.2)	51 (31.5)	
Smoking, n (%)				0.285
	1	72 (44.4)	63 (38.9)	
	2	63 (38.9)	77 (47.5)	
	3	27 (16.7)	22 (13.6)	
Statin, n (%)				0.265
	0	75 (46.3)	59 (36.4)	
	1	87 (53.7)	103 (63.6)	
RACE_BLACK, n (%)				0.460
	0	119 (73.5)	113 (69.8)	
	1	43 (26.5)	49 (30.2)	
SUB_CVD, n (%)				<0.001
	0	129 (79.6)	101 (62.3)	
	1	33 (20.4)	61 (37.7)	

DBP, diastolic blood pressure; HDL, high-density lipoprotein; BMI, body mass index.

The LASSO regression identified 14 key variables. The LASSO ranking of these variables was (in descending order): age, SUB_CLINICALCVD, Glucose, N_AGENTS, serum creatinine, RACE_BLACK, INCLUSIONFRS, UMALCR, triglycerides, BMI, SUB_CKD, SMOKE_3CAT, RACE4, DBP. These variables were important features that significantly influenced the target variable during model construction.

The mean cross-validation AUC of robust models differed from the original 60/40 validation AUC by <0.03, specifically: GBM: cross-validation AUC mean 0.7749 (standard deviation 0.0582), original validation AUC 0.7632, with a difference of merely 0.0117. AdaBoost: cross-validation AUC mean 0.7433 (standard deviation 0.0803), original validation AUC 0.7434, nearly identical. RF: Cross-validation AUC mean 0.7197 (standard deviation 0.0799), original validation AUC 0.7521, difference 0.0324 (approaching the 0.03 threshold). The mean and standard deviation of the training set’s 10-fold cross-validation model performance are all shown in Table 2.

**Table 2.** **The performance of the training set’s repeated 10-fold cross-validation**.

Model	AUC (mean)	AUC (std)	Accuracy (mean)	Accuracy (std)	Precision (mean)	Precision (std)	Sensitivity (mean)	Sensitivity (std)	Specificity (mean)	Specificity (std)
SVM	0.5178	0.2694	0.5668	0.0414	0.7000	0.4583	0.1044	0.0922	1.0000	0.0000
Adaboost	0.7433	0.0803	0.6605	0.0808	0.6580	0.0758	0.6089	0.1350	0.7100	0.0539
MLP	0.4760	0.1629	0.5213	0.0487	0.3267	0.3596	0.0989	0.1159	0.9200	0.0748
RF	0.7197	0.0799	0.6439	0.0555	0.6350	0.0599	0.6289	0.1119	0.6600	0.0800
NB	0.7538	0.0723	0.6439	0.1019	0.7588	0.2131	0.4122	0.1559	0.8600	0.1200
LR	0.7487	0.0802	0.6955	0.0762	0.7119	0.1240	0.6489	0.1159	0.7400	0.1200
GBM	0.7749	0.0582	0.6908	0.0457	0.7238	0.1112	0.6278	0.1239	0.7500	0.1204

### 3.2 Comparison of Model Performance

**Fig. 4.**
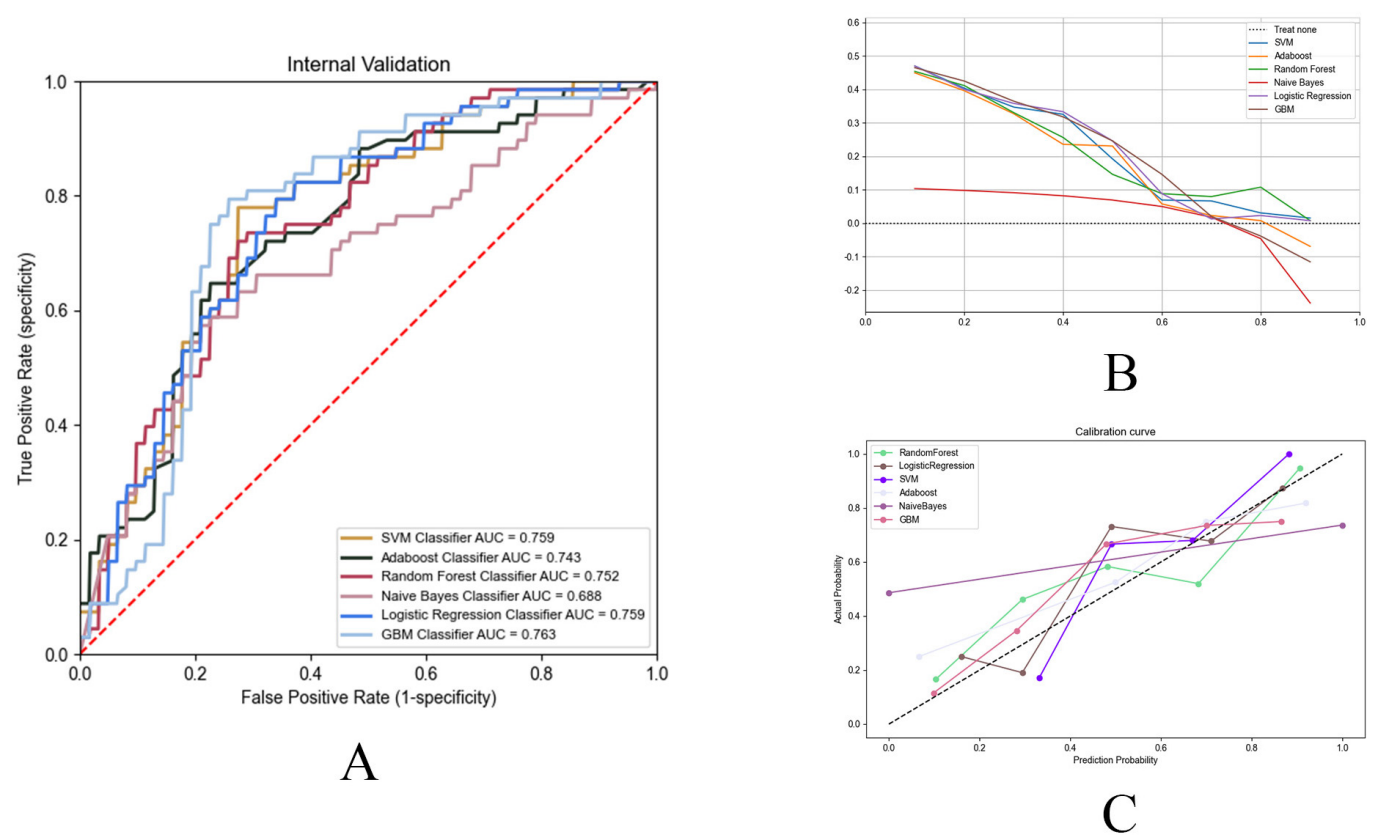
**Performance evaluation of machine learning prediction models**. (A) The receiver operating characteristic (ROC) curves of the top six best-performing ML models. ML, machine learning. (B) DCA curves of the top six best-performing ML models. (C) Calibration curves of the top six ML models.

Seven ML models leveraging the SPRINT database were constructed and compared (Table 3). Among these six algorithms, the GBM (AUC = 0.763, accuracy = 0.731, precision = 0.770) and RF (AUC = 0.752, accuracy = 0.708, precision = 0.734) models demonstrated similarly high performance in predicting HF. In contrast, the MLP model exhibited the lowest performance, with an AUC of 0.546, accuracy of 0.523 and precision of 0.523 (Fig. 4A, Table 3). The GBM model demonstrated superior predictive performance compared to the RF model, although this difference did not reach statistical significance (*p* = 0.425). Notably, the GBM model outperformed other models in both DCA and calibration curves, indicating superior clinical utility and reliability (Fig. 4B,C). Consequently, GBM was selected as the final model.

**Table 3.** **Performance comparison of GBM and six other machine learning algorithms**.

Model	AUC	AUC (95% CI)	AUC (95% CI)	Accuracy	Precision	F1_Score	Sensitivity
		lower	upper				
SVM	0.759	0.675	0.846	0.515	1.000	0.137	0.074
AdaBoost	0.743	0.666	0.838	0.692	0.706	0.706	0.706
MLP	0.546	0.447	0.643	0.523	0.523	0.687	1.000
RandomForest	0.752	0.667	0.827	0.708	0.734	0.712	0.691
NB	0.688	0.592	0.795	0.546	0.737	0.322	0.206
LR	0.759	0.674	0.831	0.677	0.717	0.672	0.632
GBM	0.763	0.676	0.840	0.731	0.770	0.729	0.691

Consequently, GBM was selected as the final model. The DCA and calibration curves for all models are presented in Fig. 4B,C.

### 3.3 Model Interpretation

**Fig. 5.**
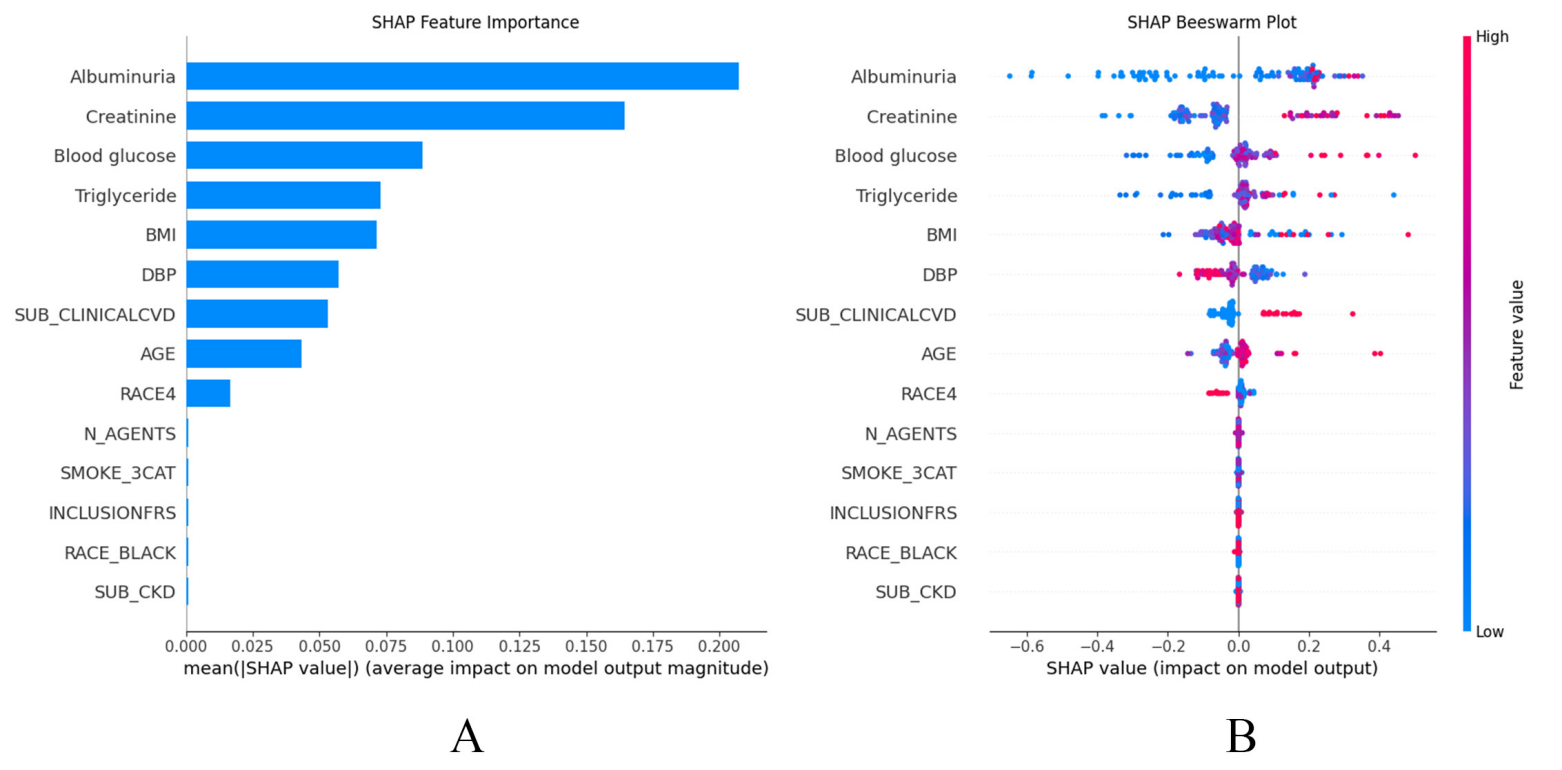
**SHAP summary plots**. (A) SHAP summary bar plot. Clinical features are ranked based on importance for predicting HF, with longer bars representing greater influence on model output. (B) SHAP summary dot plot. Individual contributions of clinical features to the incident HF predictive model. Each point represents a single observation, with its position indicating the impact of that feature on risk prediction. The color of each point reflects the feature’s value.

**Fig. 6.**
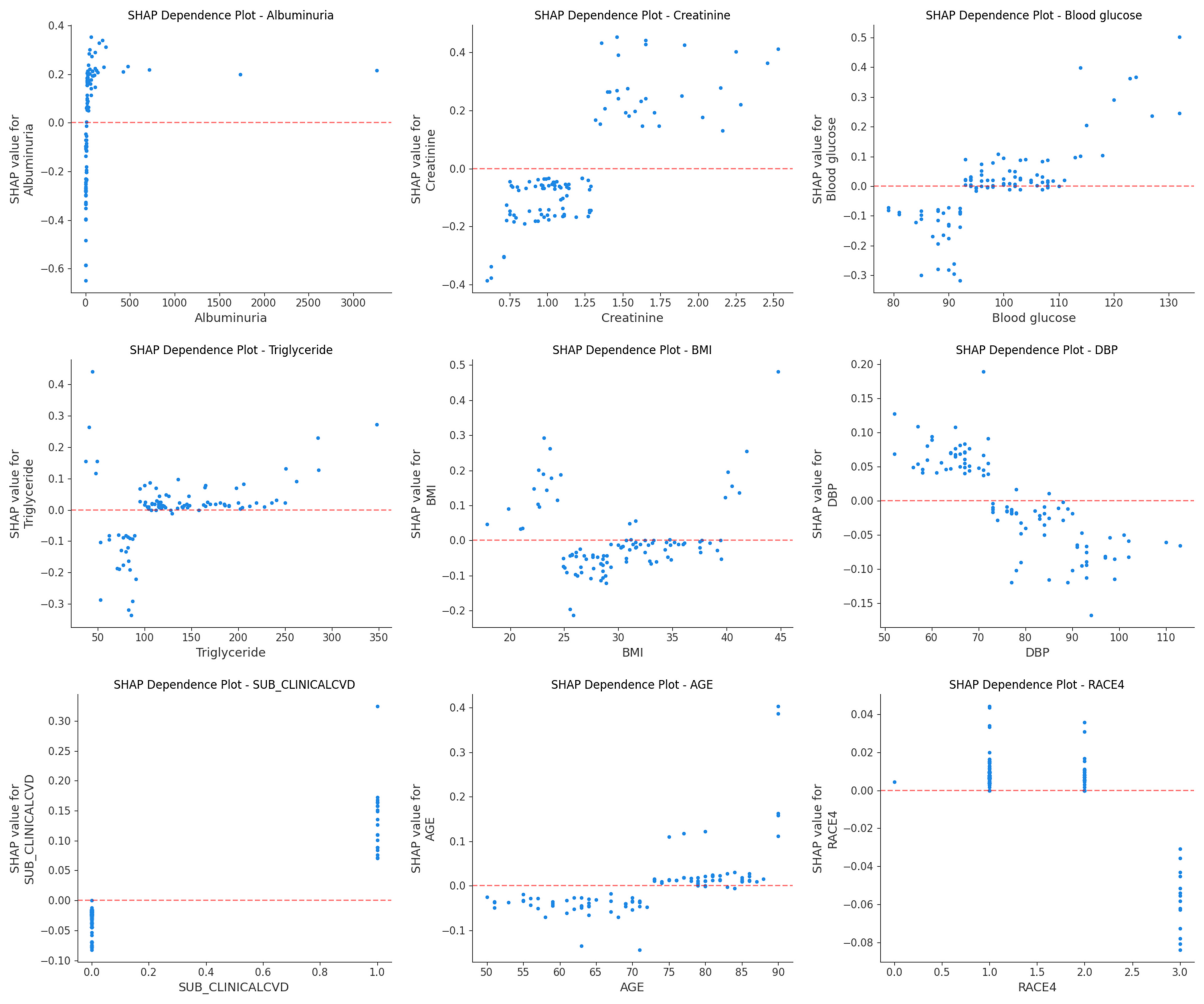
**SHAP dependence plot**. Each plot reflects the contribution value of a feature (top nine) to SHAP, with SHAP = 0 determining the threshold. A point represents the individual’s actual value.

Since clinicians may be reluctant to adopt a prediction model that lacks transparency and interpretability, the SHAP method can provide both global and local interpretability, which could describe the overall function (global) and reflect individual case (local). As shown in the SHAP summary plots (Fig. 5A,B), the contributions of the features to the model are presented in descending order. Notably, the GBM model exhibited a high degree of sensitivity to variables such as UMALCR, creatinine, blood glucose, triglyceride, BMI, DBP, history of CVD, age, and RACE4 (Fig. 5A). Additionally, the SHAP dependence plot facilitates understanding of how a single feature affects the output of the prediction model. Real values with corresponding SHAP values greater than zero indicate a positive class prediction, which in this case represents a higher risk of HF, while a negative SHAP value indicates a lower risk of HF. Therefore, the feature value corresponding to SHAP = 0 is used as the feature threshold. Fig. 6 shows the real values versus SHAP values for these nine features. For instance, patients with an UMALCR ≥8.38 mg Urine Alb / (g Creat * 0.01), mg/g Cr or Creatinine ≥1.3 mg/dL or Blood glucose ≥108 mg/dL or Age ≥80 years or Triglyceride ≥107 mg/dL or DBP <88 mmHg had SHAP values above 0, pushing the model’s prediction toward the HF class. In addition, BMI values <24.01 or ≥33.25 also increased the risk prediction for HF.

## 4. Discussion

We developed a novel ML model to predict HF risk for patients with hypertension, and provided feature importance and corresponding thresholds to aid in making better clinical decisions. The simplified model was derived from the evaluation of 28 candidate variables in the SPRINT cohort, comprising individuals free of HF at baseline. It exhibited good performance in the internal validation cohort. Taken together, these findings may inform the development of improved HF monitoring strategies in patients with hypertension.

Although several predictive models can identify HF [11, 23, 24], none to our knowledge have been specific to patients with hypertension. The ML approach offers distinct advantages over conventional risk prediction methods, as it can process extensive, high-dimensional time-to-event datasets without requiring assumptions of normality, linearity in risk estimation, or concerns about model overfitting. Compared to unsupervised machine learning, supervised machine learning offers greater predictive value and better reflects the relationship between features and outcomes. A substantial body of evidence demonstrates that hypertension is closely associated with the development of HF [3, 25, 26, 27]. Consequently, employing appropriate methodologies to predict HF risk within the hypertensive population is critically important. Indeed, in the SPRINT cohort, ML methods demonstrated good risk prediction performance and addressed the existing gap in forecasting HF risk among individuals with hypertension.

A lack of established guidelines or consensus on feature selection in predictive modeling creates uncertainty about the optimal number of features to include. While expanding the feature set can improve the model’s informational richness, excessive inclusion may reduce its clinical applicability. Furthermore, integrating non-causal features may negatively impact predictive performance [28]. To address this, we first identified the required clinical variables through expert screening and employed the LASSO method to determine the 14 most influential features. The SHAP method is also employed for feature selection. Ultimately, we constructed a machine learning prediction model based on the GBM method using the filtered feature variables, demonstrating robust predictive capability for heart failure.

Our risk prediction model enhances understanding of the broad mechanistic contributions to HF development among patients with hypertension, primarily through the use of the SHAP method. While clinical risk factors such as creatinine, blood glucose, triglyceride, BMI, and DBP are commonly used in practice, we assessed their relative importance in predicting HF and showed that their associated risk varies along a continuum. Notably, many of these markers may be clinically silent and not classified as abnormal in a substantial proportion of patients [29, 30]. Furthermore, our findings confirmed the involvement of multiple physiological systems, including the renal system (e.g., albuminuria), in the transition from cardiometabolic disorders to HF. The kidneys play a crucial role in this progression, with proteinuria and creatinine identified as the most and second most influential features, respectively. Numerous studies have now demonstrated that CKD frequently coexists with heart failure [31, 32]. Research by Zhang *et al*. [33] indicates that CKD can lead to gut microbiota dysbiosis, wherein Escherichia coli modulates the production of sulphate indophenol, ultimately resulting in the development of heart failure (via the AHR-CYP1B1 pathway). Moreover, CKD patients frequently experience sodium retention, activation of the renin-angiotensin-aldosterone system, and sympathetic nervous system stimulation, which collectively elevate blood pressure. This creates a vicious cycle that increases the risk of heart failure. These mechanisms also corroborate our findings. Therefore, beyond established risk factors for HF, our data help identify additional, non-hypothesis-driven markers for predicting future HF risk. Importantly, the previously supervised ML studies have not been developed the HF predicting model for hypertension population, most of them are designed for the individuals with normal blood pressure. In contrast, our study specifically identified and ranked risk factors associated with HF in patients with hypertension.

Our model possesses a certain degree of external generalizability. Firstly, our model exhibits outstanding stability. In addition to SVM and MLP, the standard deviation of AUC for other models was <0.0805, according to Table 2 (minimum: GBM 0.0582, maximum: SVM 0.2694); The clinical core model (GBM) exhibited a standard deviation of 0.0582 and a standard deviation of accuracy of 0.0457. These values fall well below the threshold for “acceptable stability” (standard deviation ≤0.05–0.10), indicating minimal model fluctuation across different data subsets and no risk of overfitting. Secondly, the results demonstrate high consistency: the AUC difference between cross-validation and the original 60/40 validation was consistently <0.035, confirming that the original single validation outcome was not distorted by data partitioning bias and thus possesses reliable reference value. Moreover, both the DCA curve and calibration curves demonstrate that the model possesses sound clinical decision-making value. However, as the SPRINT cohort excluded individuals with diabetes and incorporated an intensive blood pressure lowering treatment, this study offers limited predictive value for hypertensive patients with diabetes. Nevertheless, it provides robust predictive utility for populations undergoing intensive blood pressure lowering therapy.

## 5. Limitations

We acknowledge several limitations of this study. The SPRINT study concluded in 2015, and the risk factors for HF may have varied over the last decade. Additionally, the absence of electrocardiogram (ECG) data limited our ability to integrate ECG findings into the predictive modeling process. Similarly, Certain other indicators potentially affecting heart failure (such as cardiorespiratory fitness levels) are not available in SPRINT trial. Moreover, this study did not differentiate between HF with reduced ejection fraction (HFrEF) and HF with preserved ejection fraction (HFpEF). Further research is warranted to establish population-specific risk scores for predicting the likelihood of HFrEF and HFpEF in both the general population and individuals with hypertension. The lack of ECG, N-terminal pro-brain natriuretic peptide (NT-proBNP), and echocardiographic indicators, such as left ventricular ejection fractions, limits the clinical applicability of our model. Finally, the use of random undersampling may result in information loss, potentially diminishing the representativeness of the majority-class samples. Therefore, we recommend that future research incorporate multimodal data to improve predictive performance and clinical utility.

## 6. Conclusion

We successfully developed an explainable ML model to predict HF in patients with hypertension using data from the SPRINT study. The GBM model has demonstrated relatively good predictive value among all ML models. This approach not only enhances the efficiency of HF risk assessment, but also supports personalized medical care. Furthermore, ML-based methods outperform traditional risk prediction models by both validating established biomarkers and identifying novel subclinical markers, providing critical insights into HF risk among patients with hypertension.

## Data Availability

Data from this study were from the SPRINT dataset, available at National Heart, Lung, and Blood Institute BioLINCC data repository. Researchers can apply for access to this database by visiting https://biolincc.nhlbi.nih.gov/home/.

## Abbreviations

HF, heart failure; ML, machine learning; SHAP, SHapley Additive explanation; SPRINT, Systolic Blood Pressure Intervention Trial; CKD, chronic kidney disease; SVM, support vector machine; Adaboost, adaptive boosting; NB, naive Bayes; LR, logistic regression; GBM, gradient boosting machine; RF, random forest; MLP, multilayer perceptron; DCA, decision curve analysis; CVD, cardiovascular disease; ECG, electrocardiogram; INCLUSIONFRS, Framingham 10-year CVD risk >15%; SBP, systolic blood pressure; DBP, diastolic blood pressure; N_AGENTS, number of anti-hypertensive medications prescribed; eGFR, epidermal growth factor receptor; RACE_BLACK, race (Black, African-American); SUB_CLINICALCVD, subgroup with history of clinical CVD; Race4, race (White, Hispanic, Black, other); HDL, high-density lipoprotein; UMALCR, Urine Albumin/Creatinine ratio - mg Urine Alb / (g Creat * 0.01); BMI, body mass index; SUB_CVD, subgroup with history of clinical/subclinical CVD; SUB_CKD, subgroup with CKD (eGFR <60 mL/min/1.73 m^2^); SMOKE_3CAT, never smoker, former smoker, current smoker.
